# Electroencephalogram Application for the Analysis of Stress Relief in the Seasonal Landscape

**DOI:** 10.3390/ijerph18168522

**Published:** 2021-08-12

**Authors:** Yuting Wang, Ming Xu

**Affiliations:** 1 Henan Key Laboratory of Earth System Observation and Modeling, Henan University, Kaifeng 475004, China; wyt2019@vip.henu.edu.cn; 2 College of Geography and Environmental Science, Henan University, Kaifeng 475004, China; 3 Key Laboratory of Geospatial Technology for the Middle and Lower Yellow River Regions, Henan University, Ministry of Education, Kaifeng 475004, China

**Keywords:** neuroscience, electroencephalogram, stress recovery, landscape, season, cultural ecosystem services

## Abstract

This study proposes an integrated approach to assess the psychological and physiological responses of people in natural seasonal landscapes. The questionnaire of restoration outcomes scale (ROS), willingness to visit (WTV), cultural ecosystem services (CES) cognitive classification, and the neuroscientific technique based on electroencephalogram (EEG) measurements were applied. The effects of different landscapes on human perception were studied by comparing the EEG data of different landscape types and different seasons. The coupling relationship between EEG data and stress recovery was also examined. The results showed the following: First, there was a significant difference between the winter landscape and the summer natural landscape. Second, only the winter landscape showed significant gender differences. Third, the values of ROS and WTV in the summer landscape were greater than those in the winter landscape. Fourth, the number of CES in the summer landscape was significantly higher than that in the winter landscape, and the number of CES in water was higher than that in the forest and grassland. Thus, brain wave data and quantified values from questionnaires including ROS, WTV, and CES showed significant seasonality. Therefore, an EEG can be used as a new, more objective tool and method for landscape evaluation and planning in the future.

## 1. Introduction

Natural landscapes have numerous benefits, including a positive impact on human health [1,2]. Traditional landscape assessment is mostly based on questionnaire surveys and self-assessment. It is hard to obtain the original data, and some personal language description errors may be included in it. Moreover, the data are subjective, difficult to quantify, and poor in controllability. In recent years, the development of neurology technology has provided measurable, objective technologies and methods for the study of brain activity and human perception of landscape environments. Some biofeedback and brain imaging devices are gradually being used to study human brain activity and human perception in landscape environments [3].

For example, measuring brain activity with various types of brain imaging equipment is an objective method to obtain physiological indicators in contact with the environment [4,5,6]. Brain imaging can measure the effects of unconscious or masked sensations [7,8]. The brain has a recognized pattern of activity for various emotional responses and alertness or resting states [9,10]. Direct evidence of the human brain has promoted new landscape research in this field. The latest EEG technology provides an opportunity for landscape research to explore the relationship between landscapes and human perception through the use of direct brain information.

Different landscape types have different effects on people’s perception, physiology, and psychology. Research in this area focuses on natural and artificial environments. Ulrich [1] divided landscape types into three categories: (1) nature with water, (2) nature dominated by vegetation, and (3) urban environments without water or vegetation. The results showed that the two categories of nature views had more positive influences on psychophysiological states than urban scenes. Moreover, Laumann et al. [11] tested the recovery quality of different environments through questionnaires to determine the restoration people felt in specific environments. Similar studies found that natural landscapes had a stronger positive effect on health than urban landscapes [12]. Tilley et al. [13] adopted emotive mobile EEG to measure the degree of excitement, participation, and frustration of the elderly in specific urban and natural environments. Most landscapes are classified as “natural” or “urban.” Furthermore, most studies have used only rough landscape categories. Few studies have found the use of subcategories in these groups [12]. Tang et al. [3] used functional magnetic resonance imaging (fMRI) to discuss the relationship between different landscape environments and human brain activity. However, the impact of different natural landscapes on people has not been discussed.

The seasonal natural landscape also has a certain effect on people’s preferences. The season depicted had a significant impact on people’s estimation of the legibility of the landscape [14]. Seasonal trees affected people’s visual preferences [15]. Seasonal changes in tree foliage enhanced the perceived restorative quality of schoolyard environments [16]. The public preferred summer water-wise landscapes [17]. Seasonal transformation had an important effect on both preference and restoration [18]. Some studies have shown seasonal landscape to have a strong influence on human beings’ psychological and physiological responses. Song et al. [19] showed that the urban park environment in winter had a relaxing effect compared with urban areas and had a positive effect on healthy individuals by measuring heart rate and heart rate variability (HRV) and using the scale method. Another study showed that there was no difference in forest bathing in the forest landscape between summer and autumn with the scale method [20]. Bielinis et al. [21] used a psychological questionnaire survey method to confirm that broad-leaved trees had greater restorative effect in winter than in spring. Some studies also described the relationship between trees with different stands, densities, and stress levels in winter based on brain–computer interfaces. The results showed that the forests with high-density conifers and low-density hardwood had a better pressure recovery effect [22].

Since Kaplan [23] proposed the theory of attention restoration (ART), the restorative effects of the natural environment have been continuously confirmed in other psychological and physiological studies. Contact with nature has been consistently associated with positive mental health [1,11,24,25,26,27,28]. Natural landscapes are believed to affect humans in many ways, including aesthetic appreciation, health, and well-being [18]. Aesthetic and emotional experiences seem to be the most important benefits that many recreationists derive from the natural environment [2,29,30].

The willingness to visit and the culture ecosystem service (CES) subcategories [31] have been used to represent people’s perceptions of different seasonal landscapes. Ecosystem services are defined as the benefits that people obtain from ecosystems and are generally classified into provisioning services, regulating services, supporting services, and cultural services [32]. CES are the non-material benefits that people gain from ecosystems through cultural heritage, spiritual enrichment, recreation and tourism, and aesthetic experiences, which refer to the nonmaterial benefits that people obtain from ecosystem [32,33] and that directly influence life quality [34].

The above studies either evaluated the landscape from the data obtained from scale scoring and questionnaire surveys or analyzed the relationship between the stress level of a single landscape or single season and human perception using neurology technology. However, there were few findings on the relationship between various seasonal landscapes and stress recovery. This research, therefore, focused on reasonably quantitative EEG technologies to study the impact of different natural landscape forms and seasons on brain activity and stress recovery. The aims of the study were as follows:

(1) The differences in seasonal natural landscape from the viewpoint of physiology and perception were explored, as well as the differences among different landscape types. Do natural landscapes in one season relieve stress more effectively than landscapes in another season, and do some landscape types have better stress recovery effects than others?
(2) The relationship between the restoration outcomes scale (ROS), willingness to visit (WTV), cultural ecosystem services (CES), and EEG data was coupled. At the same time, it provides a more objective idea and method for cultural ecosystem services evaluation.

## 2. Materials and Methods

### 2.1. Materials

**Figure 1:**
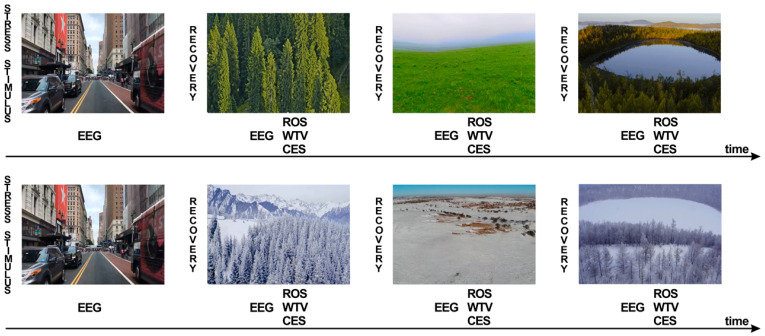
Sequence of stimuli presentation, EEG recording and questionnaire of ROS, WTV and CES.

**Figure 2:**
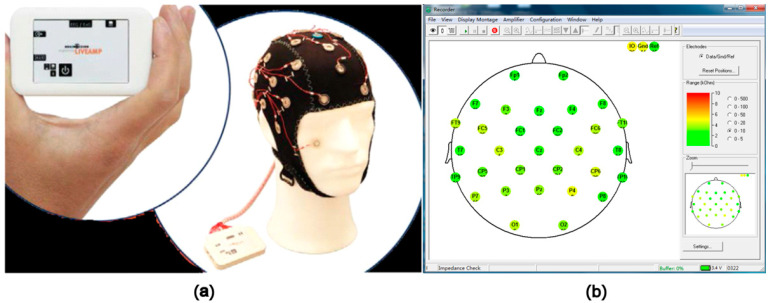
Brain Products (LiveAmp) in the experiment; (**a**) Brain Products (LiveAmp) with 32 channels; (**b**) electrode position of international 10–20 system with 32 channels.

The stimuli included seven high-definition videos, mainly including different seasons (winter and summer) and different landscape types (grassland, forest, and water), and different city settings as stress stimuli (Figure 1). The Brain Products (LiveAmp) with 32 channels was used to obtain the signals of the brain activity (Figure 2a), and the subjects were stimulated through video experience. During the experiment, conductive paste was used to reduce the scalp resistance below 5KΩ (Figure 2b). Visual stimuli were presented on a 15-inch LCD monitor. A questionnaire survey was conducted concurrently (Supplementary Materials).

### 2.2. Subjects

There were 35 healthy adult volunteers, including 17 females and 18 males aged 25 to 55, who participated in the experiment. They were healthy, right handed, and had enough sleep and good sleep quality the day before the experiment. Before the experiment, the experimental procedure was told, participants were allowed to ask questions, and a preliminary health survey was conducted to ensure that the health criteria of the test were met. Additionally, a video was played in the lab, during which the participants were asked to stay as still as possible so that interferences such as EMG did not increase. If they chose to continue, each participant had to sign an informed consent form before the test, which was approved by the Research Ethics Office. In the following analysis of the results, the participants were split into two groups by their ages. Those older than 35 were classified as the elderly group, and those younger than 35 were classified as the young group.

### 2.3. EEG Recording and Questionnaire Survey

In this study, the Active System, produced by Brain Products (LiveAmp) with 32 channels, was used to obtain signals of the brain activity (Figure 2). The original EEG data were analyzed by the EEGlab, which is the toolbox for processing continuous EEG. The EEG signals were detrended, with the average of left and right mastoids as reference. Then, eye electrical, electromyography, electrocardiography, power frequency interference, and other disturbance artifacts were removed by independent component analysis (ICA) [35,36,37]. Next, the EEG signals were segmented into contiguous 2 s windows, and any segments with the retained artifact were rejected [38]. The frequency domain features were extracted to obtain the logarithmic frequency energy values of the waves in the five frequency bands of (1–4 Hz), theta (4–8 Hz), alpha (8–13 Hz), beta (13–30 Hz), and gamma (>30 Hz) [39].

The absolute value of the EEG of various types of pictures showed that natural landscape had a certain role in the recovery of stress, the reason being that for the purpose of the stress assessment, alpha and beta waves are the most suitable indicators [6]. However, this study used short video, and alpha waves need more excitement [22,40]. Therefore, this study mainly used beta waves as an indicator of brain electrical activity.

We used the answers of ROS [22] questionnaire to show the stress resilience of different seasonal landscapes. At the same time, the answers of WTV and the number of CES subcategories [31] were used as people’s perception of different seasonal landscapes. In this study, the activities that participants chose to engage in were grouped in each landscape to reflect the different categories of CES, namely, aesthetic values, recreation (e.g., walking, horse riding, swimming, angling, and hunting), spiritual services, inspiration, sense of place, and educational values. Respondents could select several associated activities as they wished per landscape, so some landscapes fell into multiple CES engagement categories. We used the number of CES subcategories selected by subjects in different landscapes as an indicator of cultural services in this landscape [31].

The relationship between landscape and human perception was studied by comparing the EEG data of different landscape types in different seasons. The answers of ROS, WTV, and CES were used in different seasons to assess the pattern of stress recovery across different landscape types. Then, the coupling relationship between EEG data and the questionnaire responses was examined.

### 2.4. Statistical Analysis

**Figure 3:**
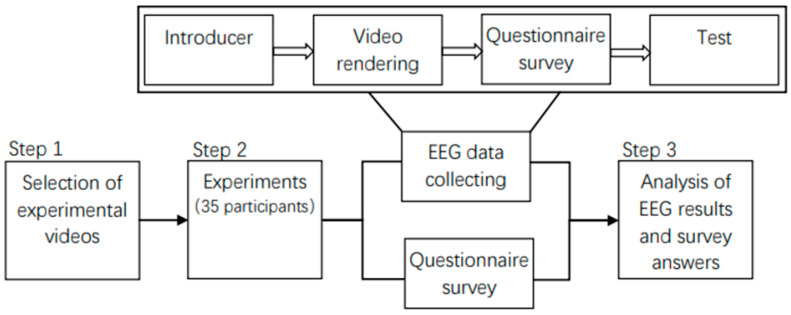
Research process of this study.

To test the differences among groups of different landscapes, seasons, genders and ages, the effects of six stimuli on brain activity were assessed with ANOVA. The answers of the ROS and the WTV and CES questionnaires were all analyzed in the same manner. At the same time, the ANOVA, Tukey’s HSD test, and the consistency of the internal answers of the ROS were also analyzed. The Pearson correlation coefficient was used to couple and verify the EEG data and the answers of questionnaire survey. All statistical analysis was performed on the R platform. The graphics were completed by Matlab2013b and Excel, and the combination of graphics was completed using CorelDRAW2018. Figure 3 displays the experimental process.

## 3. Results

### 3.1. EEG Analysis of Different Landscapes

Table 1 shows the result of two-way ANOVA, which showed the significant impact on people’s perception in landscape types, season, gender, and age in the beta waves. Besides, the interaction effect between gender and age was significant (*p* < 0.001), as well as between landscape, gender, and age (*p* < 0.05), and also between season and gender (*p* < 0.1). As the interaction effect among the factors was verified in the previous analysis, we performed a Tukey’s test to show which circumstances affect what people perceived by each factor.

#### 3.1.1. Seasonal Differences in Different Landscape

**Figure 4:**
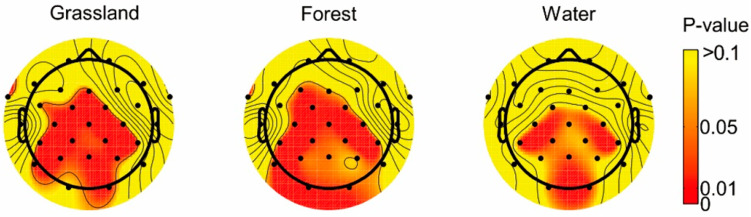
The seasonal differences under different landscape types in brain regions.

Table 2 shows the absolute beta waves of multiple electrodes in different seasons and different landscape types. The seasonal differences of the whole brain regions in each landscape type are displayed in Figure 4. For the overall summer landscape and winter landscape, seasonal differences were significant, especially in 15 electrodes: C3, C4, P3, O1, O2, P7, Cz, Pz, FC1, CP1, CP2, FC5, CP5, CP6, and FT9. The grassland landscape showed significant seasonal differences in 16 electrodes: C3, C4, P3, P4, O2, P7, Fz, Cz, Pz, FC1, FC2, CP1, CP2, CP5, CP6, and FT9. Moreover, the forest landscape showed significant seasonal differences in 17 electrodes: F3, C3, C4, P3, P4, O1, O2, P7, Cz, Pz, FC1, FC2, CP1, CP2, CP5, CP6, and FT9. The water landscape showed seasonal differences at the level of 0.05 in 6 electrodes: C4, O2, Pz, CP1, CP5, and CP6. The seasonal differences of different landscape types were mainly reflected in the common electrodes, including C4, O2, Pz, CP1, CP5, and CP6. They were mainly located in the occipital, central, and parietal regions of the brain center.

Seasonal differences for different landscape types differed in the average for the whole-brain EEG (Table 3). In the grassland landscape, the absolute beta value in winter (0.69) was higher than that in summer (0.60), and the seasonal difference was significant. In the forest landscape, the absolute beta value in winter (0.74) was higher than that in summer (0.63), and the seasonal difference was significant. In the water landscape, the beta value of winter (0.73) was higher than that of summer (0.65), and the seasonal difference was not statistically significant in the whole brain. However, there were some electrodes with significant seasonal differences in the local brain region (Figure 4). In a word, the average absolute beta value of natural landscapes in winter (0.72) was higher than that in summer (0.62).

#### 3.1.2. Gender Differences and Age Differences in Different Seasonal Landscapes

**Figure 5:**
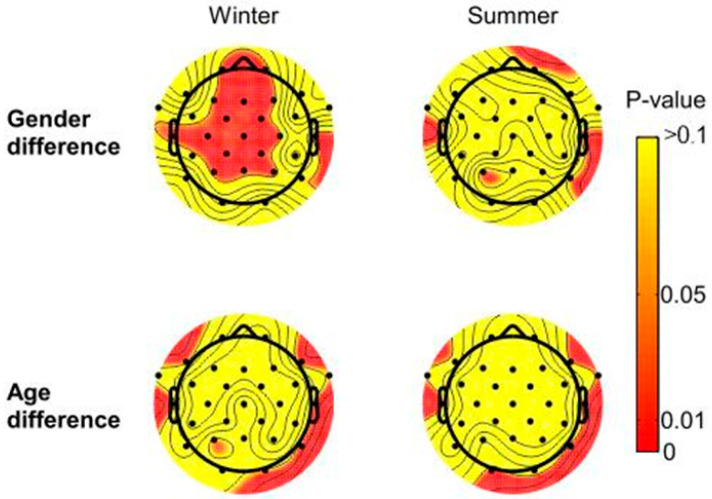
The gender difference and age difference of different seasonal landscapes in brain regions.

In terms of brain regions (Figure 5), the gender difference in the beta wave in the winter landscape was significant in 17 electrodes: Fp1, Fp2, F3, F4, C3, C4, P3, P4, T7, T8, Fz, Cz, Pz, FC1, FC2, CP1, and CP2. Moreover, the age difference was significant in F8, T7, T8, and P8. In the summer landscape, the gender difference in the of beta wave was significant in P3, T7, and T8, while the age difference was significantly reflected in O1, T7, T8, P8, and FT10.

As can be seen from Table 4, for the average of the whole brain wave, there were significant gender differences in the winter landscape but no significant gender differences in the summer landscape. Additionally, the age difference in all landscapes was not significant. In addition, the absolute beta value of women was higher than that of men in all landscapes, including the summer landscape and winter landscape (Table 4). The absolute beta value of the young group in all landscapes was higher than that of the old group (Table 4).

### 3.2. The Analysis of Questionnaire Survey

#### 3.2.1. The Analysis of ROS

Table 5 reports the means of the ROS items. The average values collected after the winter landscape were lower than that after the summer landscape. This result suggests that respondents felt more pressure recovery after watching a video showing summer natural landscape compared to the resilience after the winter natural landscape.

Before the test, the Cronbach’s α was calculated as a measure of internal consistency of the ROS questions. The Cronbach’s α of the ROS collected from the water, forest, and grassland in summer and winter were 0.88, 0.87, 0.86, 0.79, 0.91, 0.74, respectively. The ANOVA performed on the overall mean differences across treatments returned a F statistic of 6.88 and a *p*-value of 5.85 × 10^−6^, indicating that differences across the six ROS were greater than the interval variability of each scale (Table 6).

Table 6 reports that the significance of differences was tested using ANOVA and the Tuckey’s test for binary comparisons as a post hoc test. Based on the multivariate statistical analysis of social demographic factors, job, vehicle ownership, native, frequency to nature, gender, and season had significant impacts on landscape resilience, while landscape type and age had no significant impacts on landscape restoration.

The Tuckey post hoc test binary comparisons revealed that there was a significant difference in the mean answers of ROS between the winter landscape and summer landscape in the grassland and forest, while the seasonal difference of ROS was not significant in the water landscapes. However, the differences in ROS between different landscape types in the same season were not significant.

#### 3.2.2. Willingness to Visit the Different Landscapes

The investigation regarded the stated probability of visiting the proposed natural landscape to recover from stress and the maximum distance and time that respondents were willing to travel to reach a natural landscape with the shown characteristics. The mean results obtained are reported in Table 7. Among the six natural landscape types, the summer forest landscape had the largest probability of being visited and the longest time that respondents were willing to travel, followed by the summer grassland landscape, summer water landscape, winter natural landscape, and winter water landscape.

In conclusion, for the willingness to visit landscape to recover from stress, the probability of the willingness to visit the winter natural landscape was lower than or the summer natural landscape. The willingness to visit of distance and time showed the same trend.

#### 3.2.3. The Relationship among EEG Data and ROS and WTV

**Figure 6:**
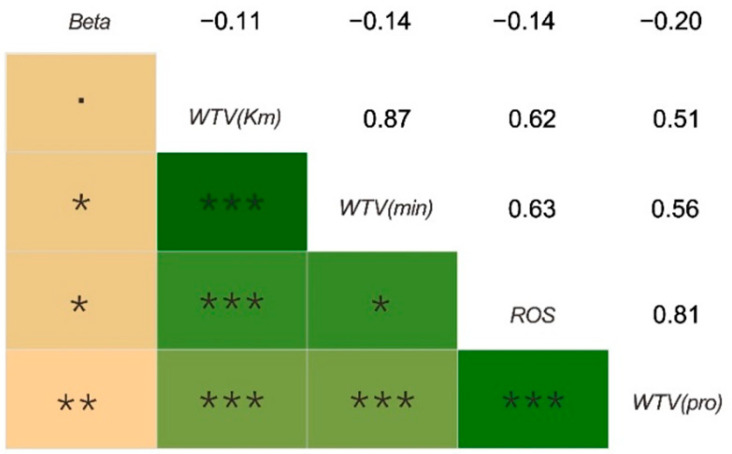
The correlation analysis between EEG data and the mean answers of ROS and WTV. Note: *** *p* < 0.001, ** *p* < 0.01, * *p* < 0.05, **•** *p* < 0.1.

We investigated whether these values were associated with EEG data across Pearson’s correlation analysis and found that the beta wave was negatively correlated with WTV and ROS. Although the correlation coefficient between the average value of the beta wave and the means of WTV data were relatively low, it was statistically significant. In addition, there was a significant positive correlation between the values of ROS and WTV (Figure 6).

### 3.3. The Analysis of Cognitive at CES

**Figure 7:**
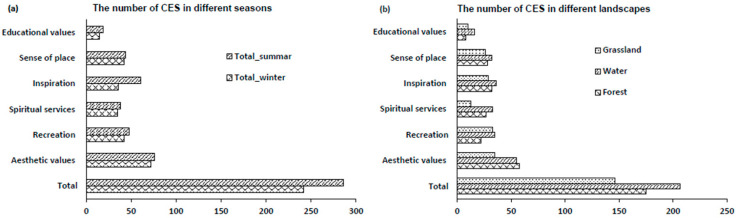
The number of CES provided in different landscapes.

From the survey and analysis results of the CES cognitive scale, we obtained the following results (Figure 7). In terms of seasonal analysis, the number of CES in the summer landscape was significantly higher than that in the winter landscape, the values of aesthetics, recreation, and spiritual enjoyment were higher than others, and the value of education was relatively low. In terms of the landscape type analysis, the number of CES in water was higher than that in the forest and grassland. However, in terms of aesthetic value, the number of CES of the forest was the highest.

## 4. Discussion

### 4.1. Differences of Physiological Responses Based on EEG Data in Different Seasonal Landscapes

There was a significant difference between the winter landscape and the summer natural landscape, and the absolute beta value of the summer landscape was significantly lower than that of the winter landscape. This result indicates that the visual stimulation of the winter landscape is much greater than that of the summer landscape and is more likely to cause anxiety or alertness. In contrast, the summer landscape brings people a feeling of relaxation, which has a positive effect on people’s health. This may be related to the theme color of the winter landscape and summer landscape, which is consistent with research about the absolute beta wave value of green being lower than that of other colors [41].

There was significant gender difference in the winter landscape and no significant gender difference in summer landscape. The female groups in all landscape types, including the summer and winter landscape, had higher absolute beta values than the male groups. The result indicates that females are more sensitive than males when they are stimulated in various landscapes, which also indicates that females are more prone to mood swings.

At the same time, there was no significant age difference in all the landscapes. As our subjects were mainly adults, future work can include a sample of more age brackets (including children) and more diverse upbringing conditions, which may help to detect the patterns of correlation between psychophysiological trends and individual characteristics, as well as focus on individual differences in natural landscape exposure.

### 4.2. The Difference of Stress Recovery Based on Questionnaire Survey in Different Seasonal Landscapes

In the ROS analysis, the season had a significant impact on the restoration of landscape pressure, while the effect of landscape type was not significant. This indicates that season is an important influencing factor in the stress restoration experiment, which has a great impact on people’s emotions. The results of ROS and WTV showed exactly the same trend. The higher the resilience, the higher the possibility of WTV, and the longer the distance and the duration of WTV. We found that beta waves were significantly negatively correlated with WTV and ROS values, while ROS and WTV values were significantly positively correlated through the analysis of the relationship among brain waves and the answers of ROS and WTV in the questionnaire. This result indicates that the beta wave can be used as a relatively objective indicator for the stress restoration of landscape to a certain extent. Our results also indicate that absolute beta waves have good statistical significance.

However, the results of studies on different stands showed that brain waves were completely unrelated to the results of WTV [22]. Therefore, seasonal factors may be a significant factor affecting the relationship between EEG and landscape stress recovery. In this study, the beta wave was significantly negatively correlated with WTV and ROS, although the correlation coefficient was relatively low. Therefore, appropriate measures should be taken to optimize the experimental design in future studies.

As the relationship between visual stimulation of color and psychological function is obvious [41,42,43,44], the leaf color of different landscape types, especially the forest, has a considerable influence on landscape aesthetics [45], and also has an important influence on people’s perception and physiological state. Therefore, future studies can quantify the vegetation cover color of different landscapes, and combine remote sensing technology to treat the landscape as a continuous variable rather than a simple seasonal binary variable, which will be an important direction for improving our research method.

### 4.3. The Seasonality of the Landscape Affects the Cultural Ecosystem Services That People Receive in It

CES, such as recreation, aesthetics, and the spirit of the natural landscape, have become an important feature of landscape research. Moreover, compared with the urban environment, exposure to the natural environment can have a positive impact on people’s psychological and physiological [1,24,27,28]. Natural landscapes are believed to affect humans in aesthetic appreciation, health, and well-being [12]. Emotional experiences and aesthetic are the most important benefits that many visitors derive from the natural landscape [2,30].

Therefore, this study also conducted a questionnaire survey on the categories of CES provided by different landscapes in different seasons. The results showed that the number of CES in summer was significantly higher than that in winter, and the value of aesthetics, recreation, and spiritual enjoyment was higher than other values. Therefore, the season is also a major factor in the ecosystem’s cultural service assessment. In this study, we also found that the seasonality of cultural ecosystem services was negatively correlated with the seasonality of the absolute beta waves. This indicates that the beta wave in the summer landscape is lower than that in the winter landscape, and the number of CES in summer is significantly higher than that in winter. This provides a way of thinking and reference for the evaluation of CES.

In this study, EEG data and the quantitative values of ROS, WTV, and CES all showed significant seasonality, and there was a positive or negative correlation between them. Therefore, in future work, EEG can be used as a new and more objective tool and method for landscape evaluation and planning, as well as a method of reference for related research, such as the evaluation of CES.

## 5. Conclusions

This paper provides a method that combines landscape resilience with brainwave analysis. The main innovation of the study was the simultaneous analysis of two variable (the landscape types and season) to evaluate natural landscape based on EEG technology from the perspective of complex systems. We also clarified the location in brain of season difference in different season landscapes, which were found to be important to model the stress recovery capacity in different landscapes.

The results confirmed the following points. First, there was a significant difference between the winter landscape and the summer natural landscape, and the absolute beta value of the summer landscape was significantly lower than that of the winter landscape. In all landscape types, the seasonal differences were concentrated on the electrodes of C4, O2, Pz, CP1, CP5, and CP6, which were located in the parietal cortex and the right occipital lobe. Second, only the winter landscape showed significant gender differences. There was no statistically significant difference in age for all the landscape, and there were only age differences on fewer electrodes. Third, the stress recovery value of the summer landscape was greater than that of the winter landscape, which showed statistically significant difference. In terms of landscape types in the same season, the stress recovery value of the forest was the highest, followed by water, and finally the grassland, but there was no statistical difference among them. Fourth, in terms of seasonal analysis, the number of CES in the summer landscape is significantly higher than that in the winter landscape. In terms of landscape type analysis, the number of CES in water was higher than that in the forest and grassland.

## Figures and Tables

**Table 1:** The ANOVA of beta waves.

Factor	Df	Sum Sq	Mean Sq	F Value	Pr (>F)
Landscape type	2	0.17	0.09	8.63	0.00 **
Season	1	0.38	0.38	37.85	0.00 ***
Gender	1	0.19	0.19	18.83	0.00 ***
Age	1	0.07	0.07	7.22	0.01 **
Landscape type: season	2	0.00	0.00	0.14	0.87
Landscape type: gender	2	0.01	0.01	0.58	0.56
Season: gender	1	0.03	0.03	3.10	0.08 ^•^
Landscape type: age	2	0.04	0.02	2.04	0.13
Season: age	1	0.01	0.01	0.75	0.39
Gender: age	1	1.36	1.36	136.88	0.00 ***
Landscape type: season: gender	2	0.01	0.00	0.44	0.64
Landscape type: season: age	2	0.02	0.01	0.95	0.39
Landscape type: gender: age	2	0.08	0.04	3.89	0.02 *
Season: gender: age	1	0.01	0.01	0.58	0.44
Landscape type: season: gender: age	2	0.02	0.01	1.21	0.30
Residuals	186	1.85	0.01		

Note: *** *p* < 0.001, ** *p* < 0.01, * *p* < 0.05, ^•^ *p* < 0.1.

**Table 2:** The absolute beta waves in different seasonal landscapes and different landscape types.

Electrode	WG	WF	WW	SG	SF	SW
Fp1	1.13 ± 0.42	1.19 ± 0.49	1.08 ± 0.48	0.87 ± 0.37	0.93 ± 0.46	1.08 ± 0.52
Fp2	1.20 ± 0.55	1.37 ± 0.62	1.30 ± 0.62	0.99 ± 0.45	1.14 ± 0.54	1.27 ± 0.63
F3	0.77 ± 0.19	0.88 ± 0.2	0.84 ± 0.23	0.67 ± 0.16	0.73 ± 0.23	0.75 ± 0.22
F4	0.80 ± 0.21	0.90 ± 0.24	0.92 ± 0.31	0.72 ± 0.19	0.77 ± 0.24	0.82 ± 0.29
C3	0.66 ± 0.13	0.68 ± 0.13	0.67 ± 0.13	0.54 ± 0.11	0.55 ± 0.14	0.58 ± 0.15
C4	0.67 ± 0.11	0.72 ± 0.14	0.69 ± 0.15	0.57 ± 0.11	0.60 ± 0.14	0.59 ± 0.14
P3	0.64 ± 0.12	0.65 ± 0.13	0.65 ± 0.12	0.53 ± 0.09	0.56 ± 0.14	0.56 ± 0.13
P4	0.65 ± 0.11	0.69 ± 0.12	0.67 ± 0.14	0.56 ± 0.10	0.60 ± 0.12	0.58 ± 0.12
O1	0.72 ± 0.13	0.78 ± 0.16	0.74 ± 0.18	0.62 ± 0.17	0.63 ± 0.15	0.61 ± 0.15
O2	0.69 ± 0.11	0.72 ± 0.12	0.69 ± 0.12	0.57 ± 0.12	0.62 ± 0.15	0.59 ± 0.14
F7	0.64 ± 0.13	0.67 ± 0.12	0.70 ± 0.19	0.57 ± 0.12	0.60 ± 0.12	0.64 ± 0.17
F8	0.68 ± 0.16	0.76 ± 0.21	0.77 ± 0.23	0.62 ± 0.16	0.69 ± 0.15	0.69 ± 0.22
T7	0.58 ± 0.13	0.59 ± 0.12	0.61 ± 0.13	0.56 ± 0.19	0.57 ± 0.16	0.54 ± 0.15
T8	0.62 ± 0.20	0.69 ± 0.25	0.70 ± 0.28	0.62 ± 0.23	0.64 ± 0.25	0.65 ± 0.28
P7	0.54 ± 0.08	0.58 ± 0.10	0.55 ± 0.11	0.46 ± 0.10	0.48 ± 0.10	0.50 ± 0.10
P8	0.60 ± 0.10	0.64 ± 0.15	0.64 ± 0.16	0.54 ± 0.15	0.55 ± 0.11	0.57 ± 0.18
Fz	0.76 ± 0.14	0.82 ± 0.17	0.79 ± 0.20	0.64 ± 0.13	0.70 ± 0.19	0.71 ± 0.20
Cz	0.75 ± 0.14	0.78 ± 0.18	0.76 ± 0.17	0.62 ± 0.12	0.66 ± 0.16	0.65 ± 0.15
Pz	0.68 ± 0.12	0.71 ± 0.15	0.69 ± 0.13	0.58 ± 0.10	0.61 ± 0.14	0.60 ± 0.12
FC1	0.73 ± 0.12	0.77 ± 0.15	0.75 ± 0.16	0.60 ± 0.12	0.65 ± 0.16	0.65 ± 0.16
FC2	0.73 ± 0.12	0.77 ± 0.14	0.75 ± 0.17	0.62 ± 0.11	0.66 ± 0.16	0.66 ± 0.15
CP1	0.68 ± 0.13	0.71 ± 0.16	0.70 ± 0.14	0.56 ± 0.11	0.60 ± 0.15	0.59 ± 0.14
CP2	0.69 ± 0.13	0.73 ± 0.16	0.70 ± 0.15	0.58 ± 0.12	0.62 ± 0.15	0.60 ± 0.14
FC5	0.67 ± 0.17	0.70 ± 0.11	0.71 ± 0.14	0.58 ± 0.13	0.61 ± 0.14	0.65 ± 0.17
FC6	0.65 ± 0.14	0.71 ± 0.15	0.73 ± 0.16	0.64 ± 0.10	0.67 ± 0.13	0.66 ± 0.13
CP5	0.56 ± 0.07	0.57 ± 0.10	0.56 ± 0.09	0.45 ± 0.09	0.46 ± 0.11	0.48 ± 0.12
CP6	0.59 ± 0.10	0.63 ± 0.13	0.61 ± 0.13	0.51 ± 0.11	0.53 ± 0.12	0.52 ± 0.12
FT9	0.49 ± 0.08	0.51 ± 0.10	0.52 ± 0.11	0.42 ± 0.10	0.44 ± 0.10	0.45 ± 0.11
FT10	0.54 ± 0.12	0.57 ± 0.13	0.57 ± 0.16	0.48 ± 0.13	0.50 ± 0.13	0.49 ± 0.12

Note: WG, WF, and WW indicate, respectively, grass, forest, water in winter; SG, SF, SW indicate, respectively, grass, forest, water in summer. Mean ± SD.

**Table 3:** The seasonal differences under different landscape types in the whole brain.

Variable	Grass	Forest	Water	Mean
Winter	0.69 ± 0.11	0.74 ± 0.12	0.73 ± 0.15	0.72 ± 0.13
Summer	0.60 ± 0.11	0.63 ± 0.14	0.65 ± 0.16	0.62 ± 0.13
Winter vs. Summer	0.02 *	0.01 *	0.14	0.03 *

Note: Mean ± SD, * *p* < 0.05.

**Table 4:** The gender difference and age difference of different seasonal landscape in the whole brain.

Variable	Gender Difference			Age Difference		
	Male	Female	Male vs. Female	Old	Young	Old vs. Young
Winter	0.69 ± 0.12	0.78 ± 0.11	0.01 *	0.71 ± 0.13	0.75 ± 0.12	0.81
Summer	0.62 ± 0.13	0.63 ± 0.14	0.99	0.61 ± 0.14	0.64 ± 0.13	0.89

Note: Mean ± SD, * *p* < 0.05.

**Table 5:** Mean answers to Restoration Outcome Scale (ROS) items after each EEG treatment.

ROS	WG	WF	WW	SG	SF	SW
Q1	3.17 ± 0.82	3.29 ± 0.89	3.20 ± 0.80	3.86 ± 0.85	3.97 ± 0.82	3.89 ± 0.90
Q2	3.23 ± 0.73	3.43 ± 0.88	3.43 ± 0.81	3.51 ± 1.01	4.09 ± 0.74	3.69 ± 0.76
Q3	2.83 ± 0.71	3.09 ± 0.82	2.94 ± 0.8	3.29 ± 0.62	3.51 ± 0.78	3.37 ± 0.81
Q4	3.23 ± 0.69	3.49 ± 0.85	3.40 ± 0.74	3.91 ± 0.66	4.00 ± 0.77	3.74 ± 0.95
Q5	3.11 ± 0.90	3.49 ± 0.89	3.31 ± 0.58	3.71 ± 0.79	3.74 ± 0.95	3.46 ± 0.74
Q6	3.49 ± 0.82	3.71 ± 0.67	3.89 ± 0.47	3.94 ± 0.68	3.97 ± 0.66	3.97 ± 0.45
Total6	3.18 ± 0.51	3.41 ± 0.69	3.36 ± 0.48	3.70 ± 0.59	3.88 ± 0.62	3.72 ± 0.62

Note: Mean ± SD.

**Table 6:** ANOVA and Tuckey tests for difference in the ROS answers among the treatments.

ANOVA						Tuckey Test Binary Comparisons				
Factor	Df	Sum Sq	Mean Sq	F Value	Pr (>F)	Test	diff	lwr	upr	*p* adj
Job	3	7.78	2.59	13.63	0.00 ***	SG vs. WG	0.53	0.12	0.94	0.00 **
Sports categories	3	1.89	0.63	3.31	0.02 *	SF vs. WF	0.47	0.06	0.88	0.01 *
Vehicle ownership	2	1.77	0.89	4.65	0.01 *	SW vs. WW	0.32	−0.09	0.74	0.23
Native	2	2.27	1.14	5.97	0.00 **	SF vs. SG	0.18	−0.24	0.59	0.86
Frequency to nature	3	13.66	4.55	23.94	0.00 ***	SW vs. SG	−0.02	−0.43	0.39	1.00
Age	1	0.69	0.69	3.65	0.06 •	SW vs. SF	−0.20	−0.61	0.22	0.79
Gender	1	7.24	7.24	38.07	0.00 ***	WF vs. WG	0.24	−0.17	0.65	0.60
Season	2	10.15	10.15	53.36	0.00 ***	WW vs. WG	0.19	−0.23	0.60	0.83
Landscape	2	1.74	0.87	4.58	0.01 *	WW vs. WF	−0.05	−0.46	0.36	1.00
Residuals	191	36.33	0.19							
Total	5	12.06	2.41	6.88	0.00 ***					
Residuals	204	71.46	0.35							

Note: *** *p* < 0.001, ** *p* < 0.01, * *p* < 0.05, **•** *p* < 0.1.

**Table 7:** Willingness to visit (WTV) in probability, kilometers, and minutes to visit natural landscape for stress recovery.

WTV	WG	WF	WW	SG	SF	SW
WTV (pro)	3.03 ± 0.75	3.23 ± 0.84	3.34 ± 0.68	4.00 ± 0.77	4.03 ± 0.75	3.94 ± 0.68
WTV (km)	24.86 ± 25.6	30.00 ± 32.43	23.49 ± 24.79	55.57 ± 47.38	54.57 ± 45.67	35.14 ± 38.83
WTV (min)	38.29 ± 29.6	39.29 ± 24.35	31.86 ± 24.44	60.43 ± 41.02	62.71 ± 41.5	48.29 ± 38.37

Note: The (pro) indicates the likelihood that you have chosen to come to this scenario after a stressful period (e.g., study, work, exams) to relieve stress and restore mood, the (km) and (min) are respectively indicates the maximum distance (in kilometers) and the maximum time (in minutes) that someone would be willing to travel in a day to the landscape. Mean ± SD.

## Data Availability

The data presented in this study are available in insert article and Supplementary Materials here.

## Supplementary Materials

Supplementary data to this article are available online at https://www.mdpi.com/1660-4601/18/16/8522/s1.
