# Supplementary material for: Electroencephalogram Application for the Analysis of Stress Relief in the Seasonal Landscape
Source: Int J Environ Res Public Health. 2021 Aug 12;18(16):8522. doi: 10.3390/ijerph18168522 (PMC8395011; doi:10.3390/ijerph18168522)
Supplement: Supplementary file 1 [file ijerph-18-08522-s001.zip › ijerph-1300564-supplementary.pdf]

Supplementary material for the research paper

"Electroencephalogram Application for the Analysis of Stress Relief in the Seasonal Landscape"

QUESTIONNAIRE

Section A—Social Demographics (before the Video)

1. Male □ Female □
2. Age______
3. What's your occupation?
    A. Student
    B. Professional technicians
    C. Personnel of enterprises and public institutions
    D. Freelancer
4. What kind of physical exercise do you do?
    A. Competitive sport
    B. Regular sports
    C. Daily fitness
5. Do you have personal transportation?
    A. I have a private car.
    B. I can use a car owned by a family member or friend.
    C. I have an exclusive motorcycle/scooter/bike for use in the city.
6. Where are you from?
    A. Inland region
    B. Coastal region
    C. Grassland region
    D. Mountains region
7. How often do you go to natural areas such as forests and parks for recreation (e.g., hiking, walking, picnics)?
    A. Almost never (less than once a year)
    B. Rarely (1–2 times per year)
    C. Sometimes (once a month)
    D. Frequently (3–4 times per month)

Section B—THE ROS Question

All options have no right or wrong answers, and we are only interested in knowing your preferences.

How do you feel after watching the video?

	To what extent do you agree with the following sentences?	Strongly disagree	Disagree	Disagree nor not agree	Agreed	Strongly agree
1.	I feel healthy and relaxed.					
2.	I feel very calm.					
3.	I feel my pressure has been lifted and I am ready for future work.					
4.	I feel very enjoyable and comfortable.					
5.	I feel I can forget my daily troubles.					
6.	My thoughts are clear.					

Section C—Willingness to Visit—WTV

1. What is the likelihood that you have chosen to come to this scenario after a stressful period (e.g., study, work, exams) to relieve stress and restore mood? (Corresponding to scores from 1 to 5)

Very low	Low	Not high nor low	High	Very high

2. (Single option) Assuming you have easy access to the landscape, what is the maximum distance (in kilometers) that you would be willing to travel in a day to the landscape?

   A. 5 km; B. 10 km; C. 20 km; D. 30 km; E. 60 km; F. 90 km; G. 120 km +.

3. (Single option) Assuming you have easy access to the landscape, what is the maximum time (in minutes) you would be willing to travel in a day to the landscape?

   A. 10 min; B. 20 min; C. 30 min; D. 40 min; E. 60 min; F. 90 min; G. 120 min +.

Section D—Cultural Ecosystem Service (CES)

The question about CES (Multiple choices, please choose at least one)

What do you usually do in this environment?

A. Enjoy the beautiful scenery
B. Recreation (Running/walking/swimming/horse riding/fishing/fishing/boating)
C. Elevate the spiritual world
D. Release pressure and cultivate sentiment
E. Sense of local characteristics
F. Educational value
